# Colostomy Reversal following Hartmann’s Procedure: The Importance of Timing in Short- and Long-Term Complications: A Retrospective Multicentric Study

**DOI:** 10.3390/jcm11154388

**Published:** 2022-07-28

**Authors:** Marco Clementi, Renato Pietroletti, Filippo Carletti, Federico Sista, Antonella Grasso, Fabiana Fiasca, Sonia Cappelli, Andrea Balla, Vinicio Rizza, Andrea Ciarrocchi, Stefano Guadagni

**Affiliations:** 1 General Surgical Unit, San Salvatore Hospital, Department of Biotechnological and Applied Clinical Sciences, University of L’Aquila, Via Vetoio, 67100 Coppito, Italy; filippocarletti1@gmail.com (F.C.); grasso.antonella86@gmail.com (A.G.); stefano.guadagni@univaq.it (S.G.); 2 Unit of Proctology and Colorectal Surgery, Val Vibrata-Sant’Omero Hospital, Department of Biotechnological and Applied Clinical Sciences, University of L’Aquila, Via Vetoio, 67100 Coppito, Italy; renato.pietroletti@univaq.it (R.P.); vinicior1979@gmail.com (V.R.); 3 Hepatic Pancreatic and Biliary Surgical Unit, San Salvatore Hospital, Department of Biotechnological and Applied Clinical Sciences, University of L’Aquila, Via Vetoio, 67100 Coppito, Italy; silversista@gmail.com; 4 Public Health Unit, Department of Biotechnological and Applied Clinical Sciences, University of L’Aquila, Via Vetoio, 67100 Coppito, Italy; fabiana.fiasca@univaq.it; 5 Department of Surgery, IRCCS Regina Elena National Cancer Institute, 00144 Rome, Italy; sonia.cappelli@ifo.it; 6 General and Minimally Invasive Surgical Unit, San Paolo Hospital, 00053 Civitavecchia, Italy; andrea.balla@gmail.com; 7 General Surgical Unit, Maria ss. dello Splendore Hospital, 67021 Giulianova, Italy; ciarro85@hotmail.com

**Keywords:** Hartmann’s Procedure, Hartmann’s Reversal, timing, early and late complications

## Abstract

The restoration of bowel continuity following Hartmann’s Procedure (HP) has been reported hitherto with high morbidity and mortality rates. No clear guidelines exist about timing in Hartmann’s Reversal (HR), the literature data being conflicting. We have sought to investigate the effect of the interval time between HP and HR in short- and long-term HR outcomes through a retrospective study based on consecutive patients undergoing HR between 2009 and 2017 in two regional hospitals in Italy. Demographic characteristics, comorbidities, intra- and post-operative data, as well as early complications, were recorded. Long-term data were collected on the surgical site occurrences of Incisional Ventral Hernia (IVH). One hundred and five patients were recruited for the study. Late HR, female gender, and long operating time were related to the highest incidence of peri-operative complications. Patients who developed IVH had undergone HR at significantly shorter times and had a higher Body Mass Index (BMI). The timing of HR seems to be an important variable linked to the onset of early and late post-operative complications. The patients submitted to early HR show a significantly lower complication rate but, at the same time, a higher rate of IVH incidence after restorative surgery. These data, in our opinion, reflect the need for planning, where possible, an early restoration of bowel continuity after HP.

## 1. Introduction

Hartmann’s Procedure (HP) is a lifesaving surgical procedure, first described in 1921 by a French surgeon, which entails recto-sigmoid colon removal with left-side end colostomy leaving the distal rectal stump closed [1]. The procedure gained popularity in the 1970s and it is currently performed in unstable patients with left colonic obstruction, ischemia, and perforation when an immediate colorectal anastomosis is deemed unsafe [2,3]. Ideally, HP should be followed by a second operation to restore bowel continuity. However, even though poor quality of life and stoma management issues make Hartmann’s Reversal (HR) a priority for many patients, only a few of these received an intestinal restoration [4,5,6]. If HP was performed for a benign condition, 35% of the comorbidity patients remained with a permanent ostomy because of high morbidity and mortality rates related to HR [7]. Besides, more than 60% of oncological patients never underwent HR due to worse conditions, disease recurrences, or the need for further medical therapies [8,9,10].

In the absence of established guidelines, the decision whether to restore bowel continuity arises from the consensus between the surgeon and the patient after discussion on the possible benefits and risks associated with the procedure. In this perspective, young age, low American Society of Anesthesiologists (ASA) score, low comorbidity, benign disease, male gender, and high-volume surgeons are factors associated both with a better peri-operative outcome and a higher reversal rate [11,12,13,14,15,16,17,18,19]. Scientific data, focused on the effect of time from the initial procedure up to the restoration on early and late outcomes, are still lacking. Starting from this evidence, with the purpose of assessing if the interval time from HP affects the early and late complication rates of restorative surgery, a database of patients submitted to HR from 2 regional centers was retrospectively analyzed.

## 2. Patients and Methods

### 2.1. Study Design

This study is a retrospective evaluation of patients who underwent HR at the San Salvatore University Hospital of L’Aquila and at an affiliated regional center (Val Vibrata-Sant’Omero Hospital) between 2009 and 2017. The patients’ data were collected from the colorectal database available in the two recruitment centers. The decision to perform HR was based on the patients’ preference and the attending surgeons’ opinion and experience, after evaluating their clinical condition. All HRs were performed by surgeons with advanced skills in colorectal surgery.

The patients with the following characteristics were excluded from the study: cases with incomplete data collection; those aged < 18 years; with an ASA score IV; residual or progressive neoplastic disease; post-Hartmann adjuvant radiation therapy; immunosuppression condition or dialyzed. Finally, also the patients who had undergone HP for anastomotic leakage after colorectal surgery and those with evidence of Incisional Ventral Hernia (IVH) prior to HR were excluded.

The patient demographics and various clinical variables were recorded in the dataset. This included information on age, gender, ASA score, Body Mass Index (BMI), comorbidity, indications for HP, days from HP to HR, operation time, hospital stay, morbidity and mortality. The comorbidities were calculated for each patient according to the Charlson Index (CI) [20]. The operative time was calculated from the incision to the last suture. Peri-operative complications were classified according to the Clavien–Dindo (CD) scale and the Comprehensive Complication Index (CCI) [21,22]. The CCI value for each patient was calculated for analysis through the CCI calculator available at the http://www.assessurgery.com (accessed on 7 October 2021) website based on the complications previously registered according to the CD scale. Post-operative mortality was defined as a death occurred within 30 days of surgery, or during the same hospital admission, as a consequence of the operation. Anastomotic Leakage (AL) was defined by the presence of any of the following clinical or radiological criteria: the presence of peritonitis as consequence of anastomotic dehiscence; the presence of feculent substances from the pelvic drain; radiological diagnosis of intra-abdominal fluid collection with AL demonstrated by endoscopy and/or contrast study [23]. For evaluating IVH occurrence, all patients were contacted by telephone almost 24 months after the restorative surgery to schedule a visit at the outpatient clinic of one of the two recruiting centers. Patients with a follow-up of less than 24 months, or patients where follow-up data could not be collected, were not considered in the long-term outcomes. All the data were incorporated into a spreadsheet (MS Excel) for statistical analysis.

The ethical approval to conduct this study was granted by the Institutional Review Board (IRB) of each participating medical center. The IRBs, due to the retrospective nature of the study, waived the need for informed consent for the use of de-identified patient data. All procedures performed in the study were in accordance with the ethical standards of the Declaration of Helsinki. The study was reported in accordance with STROBE Recommendations [24].

### 2.2. Surgical Procedure

All surgeons involved in the study had extensive training in colorectal surgery and may be considered skilled surgeons performing regularly more than 20 colorectal resections per year. The surgical technique of colonic continuity restoration was performed in all cases with a standardized technique according to the following steps: median laparotomy; lysis of adhesions to identify the rectal stump; preparation, closure, and pulled-in abdomen of the ostomy; proximal colon stump mobilization taking down splenic flexure if necessary; end-to-end anastomosis using a transanally inserted circular stapler. In all cases, a pre-operative antibiotic prophylaxis was given, as well as a mechanical colon and rectal stump (by rectal enema) cleaning preparation. In all patients the laparotomy and the residual abdominal wall defect at the stoma site were closed by a single layer interrupted suture with a slowly absorbable suture

### 2.3. Statistical Analysis

A descriptive analysis of the sample characteristics was carried out. Categorical variables (sex, ASA, CI, history of neoplasia) were described through absolute frequencies and percentages. Continuous variables like age, BMI, days between HR and HP, Hartmann’s Reversal Time (HRT), operation time, and hospital stay were expressed as median values and Interquartile Ranges (IQRs).

For the evaluation of short-term outcomes, a CCI of 12.2, which corresponds to 2 post-operative complications of CD grade I severity, was used as the threshold between high (CCI > 12.2) and low (CCI < 12.2) CCI. For the evaluation of long-term outcomes, based on IVH occurrence, the sample was stratified into two groups that were compared to each other. The statistical significance of the pair comparisons was evaluated with χ^2^ test or Fisher’s exact test for categorical variables, and with the Wilcoxon rank-sum (Mann–Whitney) test for continuous variables. The latter was also applied to assess the differences between the median values of post-Hartmann days in the absence/presence of leakage and intra-operative complications. Spearman’s correlation was used to evaluate the relationship between CCI and post-Hartmann days and the correlation was graphically represented by a scatter plot.

Statistically significant variables in the group comparisons, based on different values of CCI or long-term complications, were introduced into multivariate logistic regression models, in order to identify independent factors associated with elevated CCI and long-term complications. Akaike Information Criteria (AIC) with backward stepwise selection of variables was used to select the best multivariate model.

The statistical analysis was carried out using the statistical package STATA/IC 15.0 (Stata Corp LP, College Station, TX, USA) and *p*-values ≤ 0.05 were considered statistically significant.

## 3. Results

**Figure 1:**
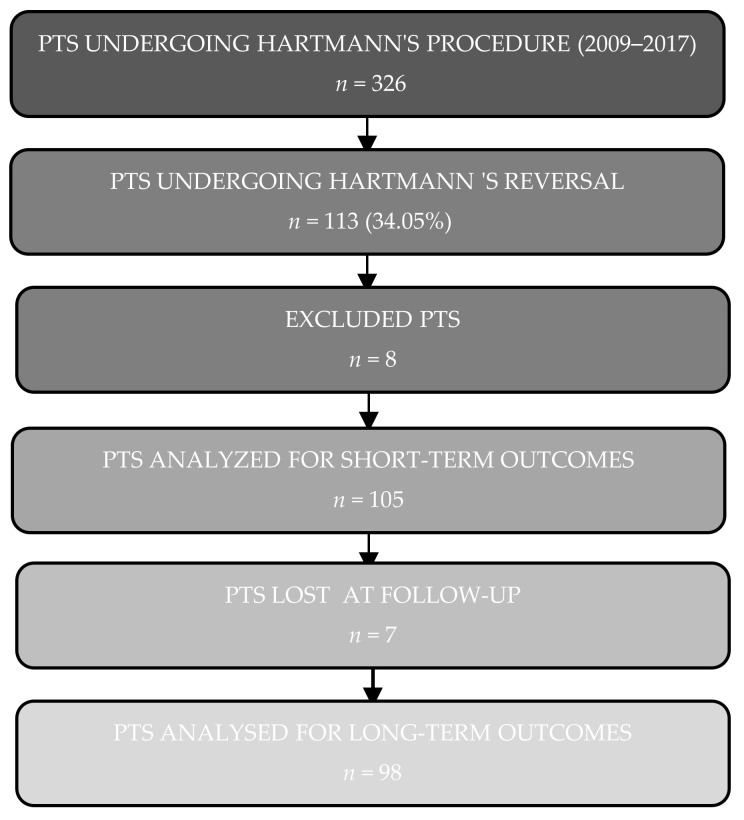
Flow diagram of patient recruitment.

During the study period, 326 patients undergoing HP were identified in our database. Of these, 113 (34.6%) had undergone HR. Eight patients were excluded from the study (4 with residual neoplastic pathology, 3 with post HP adjuvant radiation therapy, and 1 with chronic renal failure). The remaining 105 were analyzed for short-term outcomes. For 7 of these patients, no 24 months follow-up complete data were recorded, so that only 98 patients were analyzed for long-term outcomes. The flow-chart of patient selection for this study is shown in Figure 1.

### 3.1. Patients’ Characteristics

A total of 105 patients were included in the study. Of these, 44 were women (41.9%) and 61 men (58.1%). The mean age was 69 years (IQR 58–78). The distribution of ASA was as follows: ASA I-II 64 (60.95%); ASA III 41 (39.05%). The median BMI in the recruited patients was 24.47 (IQR 22.2–26.65). Diverticulitis was the most common indication to HP in 55 of them (52.4%), followed by malignancy in 23 (21.9%), and by other benign conditions in the remaining 27 (25.7%). Details of the urgent colorectal disease justifying HP are reported in Table 1.

At the time of HR, 36 (34.28%) patients had at least one comorbidity, and 6 (5.71%) had more than two comorbidities. The Charlson score was 0 in 68 of them (64.76%), 1 in 28 (26.67%), and 2 or more in 9 (8.57%). The median time between HR and HP was 152 days (IQR 62–250). The patients underwent elective surgery.

### 3.2. Short-Term Outcomes

Intra-operative complications were recorded in 10 (9.5%) patients. Small bowel perforation during adhesiolysis was the main intra-operative complication (7 cases, 6.6%). All but one of these intestinal lesions were treated with a simple suture. The median operating time was 188 min (range 90–380). Post-operative complications were detected in 61 patients (59.8%), but in 28 of these (27.4%) only CD grade I complications were recorded. Fever was the most frequent complication followed by ileus, wound infection, and prolonged parenteral nutrition. The mean value of CCI was 12.57 ± 14.15. The median time of hospitalization was 8 days (range 8–11). The intra-operative and 30-day complication details following HR are shown in Table 2.

The mean value of CCI was 12.57 ± 14.15. A CCI of 12.2, which corresponds to 2 post-operative complications of CD grade I, was used as a threshold between high (CCI > 12.2) and low (CCI ≤ 12.2) CCI. Statistically significant differences emerged for gender, with a percentage of women in the group with a more than twice higher CCI compared to that of the other group (62.16% vs. 30.88%, *p* = 0.002). Additionally, in the group with the highest CCI, HRT days, operative time, and days of hospitalization were higher than in the other group (196 vs. 119, *p* = 0.005; 200 vs. 165, *p* = 0.005; 10 vs. 8, *p* = 0.002, respectively) (Table 3).

Female sex, HRT, and operating time emerge from the multivariate logistic regression model as independent factors associated with a higher CCI (Table 4).

**Figure 2:**
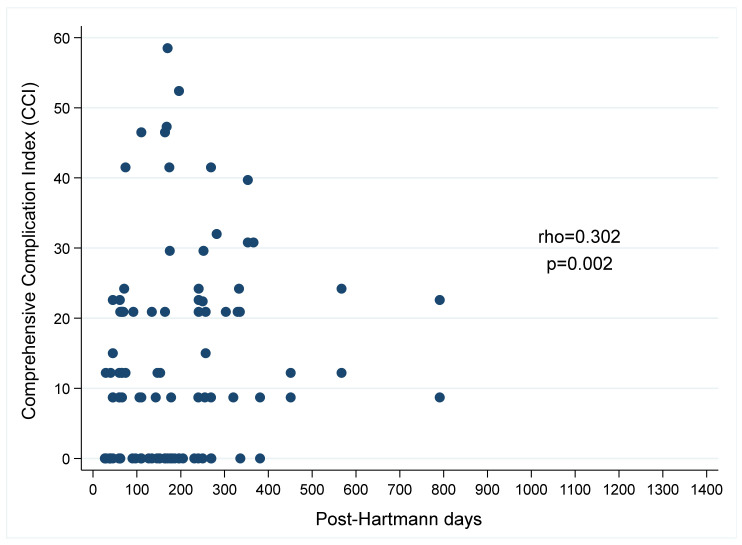
Scatter plot for the correlation between post-Hartmann days and CCI.

Spearman’s correlation shows a moderate positive significant relationship (rho = D0.302; *p* = 0.002) between CCl and HRT (Figure 2).

The median of HRT was 149 days (62–250) in the absence of leakage, and 170 (110–269) in the presence of leakage (only 7/105 patients, 6.67%), but the difference is not statistically significant (*p* = 0.386). Concerning intra-operative complications, the median HRT was 147 days (62–252) in absence of complications, and 207 (60–241) (10/105 patients, 9.52%) in the presence of complications, with a non-statistically significant difference (*p* = 0.567).

### 3.3. Long-Term Outcomes

Long-term data were collected from 95 patients. At a median follow-up of 34.9 months (range 24–56), 35 patients (36.8%) developed IVH with a median time of 195 days after HR (range 90–400). Twenty-six patients developed IVH in the previous median laparotomy, 7 patients in the prior stoma site, and in the remaining 2 cases IVH was present both at the site of the laparotomy and of the previous ostomy.

The sample was stratified into two groups based on the absence/presence of IVH. In the comparison between the two groups, statistically significant differences emerged from BMI, which was higher in patients with IVH (25.65 vs. 22.94, *p* = 0.004), HTR, higher in the absence of IVH (174 vs. 92, *p* = 0.009) and operating time, higher in the absence of IVH (190 vs. 160, *p* = 0.033) (Table 5).

BMI and HTR emerged as independent factors associated with the presence of IVH. In particular, the risk of hernia increased with the increasing of BMI (OR 1.27, 95% CI 1.10 –1.47, *p* = 0.001) and decreased with the increasing of HRT days (OR 0.99, 95% CI 0.99–0.99, *p* = 0.006) (Table 6).

## 4. Discussion

In our series, post-operative complications were detected in 60% of the cases, but in half of these only CD grade I complications were recorded. These data are in line with the overall rate of complications reported in the literature, confirming the complexity of the procedure [25,26]. A CCI of 12.2, which corresponds to 2 post-operative complications of CD grade I, was used to stratify the sample into two groups: high (CCI > 12.2) and low (CCI < 12.2) CCI. In other studies, a CCI of 26.2, which corresponds to 1 post-operative complication of CD grade III, was used as the threshold between high (CCI > 26.2) and low (CCI < 26.2) CCI [27,28]. However, these studies refer to particularly complex surgical procedures such as total radical gastrectomies or hepatectomies, in which CD complications grade III are significant in number. Our study evaluates complications of a less complex procedure, and so with a lower morbidity rate: in our series, complications higher than CD grade III were recorded only in nine patients. From this evidence the need arises to define a new cut-off of CCI at a lowest level of 12.2, corresponding to the sum of two CD grade I complications.

A late bowel restoration, female gender, long operative time, and hospital stay were related to higher peri-operative morbidity. A possible advantage of late colostomy closure is that bowel restoration is performed on a patient in better clinical and metabolic conditions, and allows a complete resolution of abdominal sepsis related to the first urgent procedure. On the other hand, early restoration may be related to less dense adhesions and may avoid the shrinkage of the rectal stump, thus reducing the difficulties of dissection and anastomosis. However, while the challenge of the shrunken rectal stump on late restoration is well documented [29], there are no studies that identify the ideal time to perform a planned relaparotomy with regard to the incidence, distribution, and severity of post-operative adhesions [30]. This essentially depends on the fact that, to date, there is no standardized system of quantification for adhesions from a previous laparotomy, so that each surgeon defines adhesions on an individual basis contingent on one’s experience.

The retrospective nature of our study does not allow an exact definition of the extent and severity of adhesions in individual procedures, but the reduction of the operative time in early recanalized patients could be an indirect scale of the difficulty encountered in adhesiolysis. Regarding intra-operative complications and anastomotic leakages, in our study the median HRT was 149 days in the absence of leakage, and 170 in the presence of leakage; median HRT was 147 days in the absence of complications, and 207 in the presence of complications. Although there was a trend toward the reduction of intra-operative complications and anastomotic leakages in patients with early recanalization, the values did not reach statistical significance.

Roe et al. first analyzed the effect of timing in HR, reporting that there was no advantage in the delay of ostomy closure [10]. Keck, a few years later, did not find a difference between the early and late groups in terms of mortality, overall morbidity, or anastomotic leakage rate, but reported, in the early group, longer bed stay and higher adhesion density grade [31]. More recently, Flemming et al. have found that the late reversal was independently associated with an increased risk of complications [32]. The analysis of patients who received colostomy reversal from 3-State Inpatient Database, represents the most extensive series available in literature [33]. The study did not detect any advantage in term of complication rate and mortality, but showed that earlier reversal was associated with a decreased length of stay and fewer readmissions. However, in this study the patients that received early restoration were the youngest and privately insured ones, leaving the possibility that these kinds of patients would have shown similar outcomes even if they had received a late restoration. All the studies cited so far, as well as our study, have the main limitation of being retrospective and multicentric with no standardized pre-operative evaluation or operative technique. In this setting, the decision to perform HR was based on the patient’s preference and the attending surgeon’s experience. In our opinion, based on the data available in the literature and in our experience, in patients with clinical or oncological conditions not excluding a new surgery, there is no reason to delay HR. Keeping this in mind, the frailty assessment in elderly patients could be considered as a useful element in predicting postoperative outcomes [34].

In our series, additional risk factors, associated with a statistically significant higher incidence of intra-operative complications, turned out to be the female gender and the long operating time. Concerning the long operating time, it is our opinion that this can be justified by the higher level of surgical complexity resulting from greater adhesions found in patients with a late bowel restoration. Regarding the female gender, no other series identified it as a risk factor for post-operative complications. This result can be attributed to the fact that, in our sample, women had a significantly higher CI than men (*p* = 0.026).

Based on the analysis of our results, if early HR contributes to reduced peri-operative complications, on the other hand, it seems to represent a risk factor for the occurrence of IVH. With a minimum follow-up of 24 months, 37% of patients in our series developed IVH. Other evidence suggests that the incidence of IVH with laparotomy or stoma closure site is high, but there are no studies that highlight the effect of time between HP and HR, and the risk of developing IVH. The high incidence of IVH after laparotomic HR could be justified by the presence of a previous laparotomy, by the higher risk related to the first urgent procedure and, finally, by the propensity to perform bowel derivation with higher surgical risk patients. In our study, statistically significant risk factors for the development of an IVH were BMI, operating time, and HRT. In particular, the risk of a hernia occurrence decreased in patients with late restoration. While a previous laparotomy and BMI are risks factors documented in the literature, there is no evidence that the time elapsed between HP and subsequent bowel restoration can play a role in the risk of developing IVH.

A previous laparotomy is a well-known risk factor affecting IVH rates [35]. Unfortunately, little is known about the effects of re-laparotomies on\wound healing in abdominal incisions. The effect of re-laparotomy timing on wound healing in an animal model was studied by Akinci et al. [36]. In this study re-laparotomies were performed 3, 5, and 30 days after primary procedures. Although the results were non-statistically significant, the authors found that early re-laparotomies did not disrupt the healing process as much as re-laparotomies performed later. However, this study analyzed very early re-laparotomies and was not conditioned by the negative effects of the urgent surgery that had led to the first laparotomy. In our experience, only 4.7% of the patients underwent HR within 30 days of the first procedure: all the patients had undergone emergency surgery and this fact was often linked to poor nutrition status and bad general condition. The possibility of delaying the restorative procedure, in our opinion, renders a better nutritional status difficult to obtain within 30 days. We may suppose that the higher incidence of hernia in patients who had undergone early recanalization is the expression of a sub-optimal nutritional status. Unfortunately, in the retrospective design of our study the nutritional status was not recorded, and therefore our hypothesis is purely speculative.

Hypothetically, the solution to the “hernia” problem on the occasion of early restoration could be overcome with the use of prosthetic material to reinforce the site of the new laparotomy, or the previous stoma. Despite the use of traditional prosthetic material being controversial in literature [37,38], a growing body of evidence supports the use of an abdominal wall reinforcement at the time of different elective and emergency colorectal procedures, to prevent ventral hernia in high-risk patients [39,40,41,42,43]. Besides, the appeal of bio-resorbable materials in prophylactic mesh augmentation in this kind of surgery, there is insufficient evidence to support their use in primary ventral hernia prevention.

Recently, various studies describing the results of HR with the laparoscopic technique have been published. The procedure has been shown to be safe, providing advantages in terms of peri-operative results with respect to the traditional approach: less pain and less hospitalization [44,45,46]. The possibility of avoiding a new laparotomy is a further advantage as it reduces the risk of IVH. However, to date, surgery performed with a minimally invasive technique represents a small part of restorative surgery, since it requires expert laparoscopy surgeons and high-volume centers. Moreover, as the laparoscopic HR does not prevent the occurrence of hernia on the site of the previous ostomy, the placement of a prophylactic mesh with the use of IPOM technique should be a safe and simple solution.

Our study has two limitations: the retrospective design and the relatively limited number of patients undergoing HR. However, to our knowledge, it is the only study in the literature that correlates the timing of HR with the risk of the appearance of IVH at a long-term follow-up.

In conclusion, this study has shown that the interval between HP and HR is an important variable linked to the onset of both early and late complications. HR performed early leads to better early post-operative outcomes, but represents a risk factor for the onset of long-term hernia occurrence. Our data should induce to consider the need for performing, where possible, an early restorative surgery. In the closure of the abdominal wall, the use of prosthetic material as well as the use of the laparoscopic technique may guarantee good prevention of the ventral hernia. However, these considerations have to be confirmed by prospective studies requiring more numerous samples.

## Figures and Tables

**Table 1:** Indications for emergency HP in patients with left side colorectal pathology.

Diagnosis	Patients, *n* (%)
Benign disease	77 (78.1)
Complicated diverticulitis	55 (52.4)
Ischemic colitis	9 (8.57)
Volvulus	5 (4.76)
Adhesion	5 (4.76)
Iatrogenic perforation	5 (4.76)
Road traffic accident	3 (2.86)
Cancer	23 (21.9)
Obstruction	18 (17.14)
Perforation	5 (4.76)

**Table 2:** Intra-operative and 30-day complications following HR.

Clavien–Dindo Grade	*n*		*n*
I	48	Fever	36
		Ileus	24
		Surgical site infection	10
		Diarrhea syndrome	4
II	33	Pneumonia	10
		Prolonged parenteral nutrition	10
		Post-operative anemia treated with transfusion	5
		Prolonged post-operative pain	4
		Heart failure	3
		Coagulopathy treated with plasma transfusion	3
		Urinary infection	1
		Atrial fibrillation	1
		Adverse drug reaction	1
		Deep surgical site infection	1
III	7	Anastomotic leakage	7
IV	3	Heart failure requiring intensive care unit	3

**Table 3:** Characteristics of the sample stratified on of the CCI ≤ 12.2 and > 12.2.

			Comprehensive Complication Index (CCI)		
		Total *n* = 105	CCI ≤ 12.2 *n* = 68 (64.76%)	CCI > 12.2 *n* = 37 (35.24%)	*p*-Value
Age, median (IQR)		69 (58–78)	73 (58–78)	67 (53–76)	0.354 *
Sex, *n* (%)					0.002 **
Male		61 (58.10)	47 (69.12)	14 (37.84)	
Female		44 (41.90)	21 (30.88)	23 (62.16)	
BMI, median (IQR)		24.47 (22.20–26.65)	24.51 (22.33–26.72)	24.14 (21.81–26.31)	0.620 *
ASA Score, *n* (%)					0.817 **
I/II		64 (60.95)	42 (61.76)	22 (59.46)	
III		41 (39.05)	26 (38.24)	15 (40.54)	
CI, *n* (%)					0.473 ***
0		68 (64.76)	47 (69.12)	21 (56.76)	
1		28 (26.67)	16 (23.53)	12 (32.43)	
≥2		9 (8.57)	5 (7.35)	4 (10.81)	
Previous cancer history, *n* (%)					0.349 **
No		82 (78.10)	55 (80.88)	27 (72.97)	
Yes		23 (21.90)	13 (19.12)	10 (27.03)	
Days between HP and HR, median (IQR)		152 (62–250)	119 (60–201)	196 (92–282)	0.005 *
Operating time (min), median (IQR)		185 (135–235)	165 (125–223)	200 (170–250)	0.005 *
Hospital stay (days), median (IQR)		8 (8–10)	8 (7–10)	10 (8–13)	0.002 *

* Wilcoxon rank-sum (Mann–Whitney) test, ** χ^2^ test, *** Fisher’s exact test, IQR: interquartile range, BMI: body mass index, CI: Charlson Index, ASA: American Society of Anesthesiologists, CCI: Comprehensive Complication Index, HP: Hartmann’s Procedure, HR: Hartmann’s Reversal.

**Table 4:** Multivariate logistic regression of the factors associated with a higher CCI.

	OR °	95% CI	*p*-Value
**Sex**			
Male ^a^	1		
Female	4.65	1.80–12.03	0.001
Days between HP and HR	1.01	1.00–1.01	0.042
Operating time	1.01	1.00–1.02	0.034
Hospital stay	1.17	0.99–1.38	0.053

° corrected for other factors in the sample, ^a^ reference category, AIC (Akaike Information Criteria) = 120, OR: odds ratio, HP: Hartmann’s Procedure, HR: Hartmann Reversal.

**Table 5:** Characteristics of the sample stratified based on the absence/presence of Incisional Ventral Hernia (IVH).

		Incisional Ventral Hernia		
	Total *n* = 98	No *n* = 63 (64.29%)	Yes *n* = 35 (35.71%)	*p*-Value
Age, median (IQR)	69 (58–78)	69 (58–78)	69 (56–76)	0.789 *
Sex, *n* (%)				1.000 **
Male	56 (57.14)	36 (57.14)	20 (57.14)	
Female	42 (42.86)	27 (42.86)	15 (42.86)	
BMI, median (IQR)	24.51 (22.20–26.65)	22.94 (21.53–26.08)	25.65 (24.14–26.79)	0.004 *
ASA, *n* (%)				0.689 **
I/II	59 (60.20)	37 (58.73)	22 (62.86)	
III	39 (39.80)	26 (41.27)	13 (37.14)	
CI, *n* (%)				0.850 ***
0	63 (64.29)	39 (61.90)	24 (68.57)	
1	26 (26.53)	18 (28.57)	8 (22.86)	
≥2	9 (9.18)	6 (9.52)	3 (8.57)	
Previous cancer history, *n* (%)				0.942 **
No	76 (77.55)	49 (77.78)	27 (77.14)	
Yes	22 (22.45)	14 (22.22)	8 (22.86)	
Days between HP and HR, median (IQR)	152 (62–252)	174 (65–270)	92 (59–176)	0.009 *
Complications, *n* (%)				0.164 **
CCI ≤ 12.2	64 (65.31)	38 (60.32)	26 (74.29)	
CCI > 12.2	34 (34.69)	25 (39.68)	9 (25.71)	
Operating time, median (IQR)	180 (135–230)	190 (150–240)	160 (125–210)	0.033 *
Hospital stay, median (IQR)	8 (7–10)	8 (8–10)	8 (7–10)	0.952 *

* Wilcoxon rank-sum (Mann–Whitney) test, ** χ^2^ test, *** Fisher’s exact test, IQR: interquartile range, BMI: body mass index, CI: Charlson Index, ASA: American Society of Anesthesiologists, CCI: Comprehensive Complication Index, HP: Hartmann’s Procedure, HR: Hartmann’s Reversal.

**Table 6:** Multivariate logistic regression of the factors associated with the presence of Incisional Ventral Hernia (IVH).

	OR °	95% CI	*p*-Value
BMI	1.27	1.10–1.47	0.001
Days between HP and HR	0.99	0.99–0.99	0.006
Operating time	0.99	0.99–1.00	0.131

° corrected for other factors in the sample, AIC (Akaike Information Criteria) = 113, OR: odds ratio, BMI: body mass index, HP: Hartmann’s Procedure, HR: Hartmann Reversal.

## Data Availability

The datasets generated during and/or analyzed during the current study are available from the corresponding author on reasonable request.
